# Community trial evaluating the integration of Indigenous healing practices and a harm reduction approach with principles of seeking safety in an Indigenous residential treatment program in northern Ontario

**DOI:** 10.1186/s12913-022-08406-3

**Published:** 2022-08-16

**Authors:** K. A. Morin, T. N. Marsh, C. Eshakakogan, J. K. Eibl, M. Spence, G. Gauthier, J. D. Walker, Dean Sayers, Alan Ozawanimke, Brent Bissaillion, D. C. Marsh

**Affiliations:** 1 Northern Ontario School of Medicine, ON Sudbury, Canada; 2 Health Sciences North Research Institute, Sudbury, Ontario Canada; 3 ICES North, Sudbury, Ontario Canada; 4 Canadian Addiction Treatment Centres, Toronto, Ontario Canada; 5 North Shore Tribal Council, Cutler, Ontario Canada; 6 McMaster University, Hamilton, Ontario Canada; 7 Batchewana First Nation, Sault Ste. Marie, Ontario Canada; 8 Sagamok First Nation, Massey, Ontario Canada; 9 Serpent River First Nation, Kenabutch, Ontario Canada

**Keywords:** Indigenous health principles, Seeking safety, Trauma, Substance use disorder

## Abstract

**Objective:**

Our primary objective was to evaluate how the Indigenous Healing and Seeking Safety (IHSS) model impacted residential addiction treatment program completion rates. Our secondary objective was to evaluate health service use 6 months before and 6 months after residential treatment for clients who attended the program before and after implementing IHSS.

**Methods:**

We observed clients of the Benbowopka Residential Treatment before IHSS implementation (from April 2013 to March 31, 2016) and after IHSS implementation (from January 1, 2018 – March 31, 2020). The program data were linked to health administration data, including the Ontario Health Insurance Plan (OHIP) physician billing, the Registered Persons Database (RPDB), the National Ambulatory Care Reporting System (NACRS), and the Discharge Abstract Database (DAD). Chi-square tests were used to compare patient characteristics in the no-IHSS and IHSS groups. We used logistic regression to estimate the association between IHSS and treatment completion. We used generalized estimating equation (GEE) regression model to evaluate health service use (including primary care visits, ED visits overall and for substance use, hospitalizations and mental health visits), Results: There were 266 patients in the no-IHSS group and 136 in the IHSS group. After adjusting for individual characteristics, we observed that IHSS was associated with increased program completion rates (odds ratio = 1.95, 95% CI 1.02-3.70). There was no significant association between IHSS patients’ health service use at time one or time two. Primary care visits time 1: aOR 0.55, 95%CI 0.72-1.13, time 2: aOR 1.13, 95%CI 0.79-1.23; ED visits overall time 1: aOR 0.91, 95%CI 0.67-1.23, time 2: aOR 1.06, 95%CI 0.75-1.50; ED visits for substance use time 1: aOR 0.81, 95%CI 0.47-1.39, time 2: aOR 0.79, 95%CI 0.37-1.54; Hospitalizations time 1: aOR 0.78, 95%CI 0.41-1.47, time 2: aOR 0.76, 95%CI 0.32-1.80; Mental health visits time 1: aOR 0.66, 95%CI 0.46-0.96, time 2: aOR 0.92 95%CI 0.7-1.40.

**Conclusions:**

Our results indicate that IHSS positively influenced program completion but had no significant effect on health service use.

**Trial registration:**

This study was registered with clinicaltrials.gov (identifier number NCT04604574). First registration 10/27/2020.

**Supplementary Information:**

The online version contains supplementary material available at 10.1186/s12913-022-08406-3.

## Introduction

It is well-known that Indigenous Peoples in Canada have experienced generations of traumatic events because of colonialism, residential school legacy, loss of land, culture, language, and freedom [1–4]. The outcome is evident in daily structural racism, family violence and lateral violence experienced by these communities [5–8]. Two major consequences of these substantial challenges are intergenerational trauma and substance use disorders (SUD). Furthermore, currently, Indigenous people are faced with the most serious health inequities in Canada; they have generally poorer physical and mental health, higher school dropout rates and illness such as diabetes, obesity, high blood pressure, depression, anxiety, and other health ailments at significantly higher rates than the general population [8–10]. These factors have strongly impacted the well-being of Indigenous people to the extent that multigenerational grief and multiple losses are associated with intergenerational trauma and substance use [3, 4, 11, 12].

Despite the high rate of issues related to mental health and substance use disorders, Marsh et al. [3, 13] and others [14, 15] have noted that Indigenous people underutilize treatment services for mental health and substance use disorders. When Indigenous people do access treatment services and residential treatment, they have high dropout rates. This is a direct consequence of a health care system that functions without a trauma-informed lens, rejects a harm reduction approach, and has limited or no understanding of Indigenous Peoples’ cultural and spiritual needs [5, 16–20].

There appears to be major gaps in residential treatment programs that offer culturally appropriate and trauma-informed treatment to Indigenous communities. This lack of treatment is evident as most Indigenous communities report a shortage of services, culturally safe treatment, and culturally informed staff [18, 21, 22]. The shortage and scarcity in treatment outcomes combined with the overrepresentation of SUD among Indigenous populations indicate that change and trauma-informed practices must be implemented and researched [5, 23, 24]. Indigenous people and communities are currently challenged by the substantial service gaps in withdrawal management, trauma treatment, aftercare, and psychiatric care [10, 25, 26]. Given that Indigenous people experience significantly higher rates of mental health problems [8] than the general population, the need for appropriate services is crucial.

Unfortunately, there is no currently available, culturally appropriate, evidence-based, integrative treatment for intergenerational trauma and SUD [8, 18, 20]. Some researchers have suggested that the key to healing, following the experience of residential school abuse and its intergenerational effects, lies in reclaiming identity [20, 27–29]. Furthermore, some authors concur that reclaiming Indigenous identity means recovering traditional values, language, beliefs, philosophies, ideologies and approaches and adapting them to the needs of today [20, 30–32]. Recovering the traditional aspects of Indigenous identity cannot be divorced from Indigenous spirituality, which is based on trust, sharing, respect, honour, and acceptance.

Indigenous cultures and communities utilize many spiritual and Indigenous healing practices (IHP) to help traumatized people heal [5, 23, 24]. The challenges Indigenous communities face to date with SUD, and the new opioid epidemic have devastated many families [18, 33, 34]. Despite these challenges, some communities have created sustaining programs in their communities. One such example is Alkali Lake (Esketemc), a Shuswap First Nation with over 35 years of experience in addiction prevention and intervention programming [9].

Mainstream residential treatment models often fail to incorporate Indigenous cultural practices into their recovery program; however, treatment programs that integrate Indigenous culture and healing practices within their program have been effective [6, 7, 20]. A few case studies report incorporating traditional healing practices into mainstream counselling and treatment interventions [5, 35, 36]. Scholars, traditional healers, and Elders encourage and utilize integration; however, some feel that these approaches require additional scientific evidence to support their use [3, 4, 13, 14, 20, 37].

In 2016, the Benbowopka Treatment Centre proposed to study a novel treatment approach which blends Indigenous Healing practices with Seeking Safety based on Dr. Teresa Marsh’s research work known as Indigenous Healing and Seeking Safety (IHSS) [2, 3, 13, 20]. This stemmed from the lack of culturally sensitive evidence-based Indigenous-focused treatment options and the growing need for effective interventions due to the opioid crisis. The IHSS model is based on an evidence-based, present-focused, coping skills counselling model for treating patients with trauma and SUD called Seeking Safety. Seeking Safety combines cognitive-behavioural therapy, interpersonal therapy, self-care skills, and case management in the treatment of trauma, SUD, or both. This IHSS model is an adaptation of Seeking Safety to treat Indigenous patients by incorporating traditional healing practices and ceremonies within the foundational Seeking Safety model.

Our primary objective was to evaluate how the implementation of IHSS impacted residential addiction treatment program completion rates. We expected that patients treated with the IHSS model would have improved completion (an increase of 10% over pre-intervention rates). Our secondary objective was to evaluate health service use 6 months before and 6 months after residential treatment in the group who attended residential treatment with and without the implementation of IHSS. We expected a more significant reduction in health system usage for addiction-related services at six-month follow-up than those treated within the abstinence-based model (decreased 10% over pre-intervention rates).

## Methods

### Setting and study design

As part of a restructuring activity, the IHSS program was initiated in 2016 at Benbowopka for Indigenous patients with a history of intergenerational trauma and SUD. Benbowopka, located in Northern Ontario, Canada, serves roughly 250 active patients across Ontario per year. Referrals to the program can come from health service providers, family or self-referrals, Indigenous communities, counselors, hospitals and Elders and healers. Before 2015, individuals on prescribed medication, including opioid agonist treatment, by their physician to address SUD or mental health challenges were not eligible for admission to Benbowopka Treatment Centre because of its abstinence-based service model. The treatment setting is described in detail in a detailed study protocol paper [18].

We conducted a pre/post-Quasi-Experimental Community trial comparing historical treatment outcomes for past Benbowopka patients to outcomes following the implementation of IHSS treatment for Indigenous patients with a history of intergenerational trauma and SUD. Our cohort included all patients of the Benbowopka Treatment Program no exclusion was applied. We conducted a quantitative analysis to assess treatment completion differences in the IHSS and no IHSS groups. We also evaluated health service use rates 6 months before and 6 months after the Benbowopka 28 day residential treatment program for the group who experienced IHSS (IHSS group) and the group who did not (no IHSS group).

### Data sources and data linkage

The Benbowopka Treatment program has high-quality data which was collected prospectively and analyzed retrospectively. Standardized assessment screening tools routinely used by the Drug and Alcohol Treatment Information System and the National Native Alcohol and Drug Abuse Program [38] were used to assess patients. We included identifiers for all program patients from April 2013 to March 31, 2020. The no IHSS intervention study window spanned over 36 months (April 1, 2013 – March 31, 2016; *n* = 343), and the IHSS intervention study window spanned the 27 months post-IHSS implementation (January 1, 2018 – March 31, 2020; *n* = 136). The post-IHSS data collection window was closed earlier than planned due to the program closing for the global COVID-19 pandemic. The cohort was shared with ICES under the protection of a comprehensive data-sharing agreement and linked to administrative data sets that defined study outcomes and covariates. The ICES is a not-for-profit research institute that manages, links and stores data to be analyzed for research under the Ontario Personal Health Information Privacy act [39]. The program data was linked using encrypted health card numbers. Therefore, only 266 patients were linked to ICES for the no IHSS group due to missing health card numbers. We then queried data 6 months before and 6 months after completing or discontinuing residential treatment at Benbowopka in the no IHSS and IHSS windows.

Program data were linked to ICES data holdings with Ontario Health Insurance Plan (OHIP) numbers. ICES databases used included the Registered Persons Database (RPDB), which provides demographic and geographic information on Ontario residents with a valid Ontario health card; the Ontario Health Insurance Plan physician (OHIP) claims database, which includes data on all physician and laboratory services; the Canadian Institute for Health Information National Ambulatory Care Reporting System, which identifies ambulatory care including visits to the emergency department; the Canadian Institute for Health Information Discharge Abstract Database, which provides data on hospital discharges and the Narcotics Monitoring System database, which collects data on dispensed prescriptions for narcotics, controlled substances and other monitored drugs. These data sets were linked with the use of unique encoded identifiers. All datasets were linked with the use of unique encrypted identifiers. ICES linkage enabled virtual follow-up of Benbowopka patients for health systems usage and substance use outcomes, as monitored by the Canadian Center for Substance Abuse. After linking, the no IHSS group had 266 patients, and the IHSS group had 136 patients.

### Study variables

The cohort included all individuals who attended the Benbowopka Treatment Program. Clients were placed into two groups based on whether they attended the treatment program when IHSS was in place (IHSS group) or before IHSS was implemented (no IHSS). All client data were anonymized at the time of data entry. Baseline covariates including age, sex, self-identified Indigenous, Indigenous status, living on-reserve, living in rural or northern areas, primary substance use, income quintile, and opioid agonist treatment (OAT) participation before attending Benbowopka Treatment Program were used to describe patients pre and post-IHSS implementation and used as adjust for potential confounding. The variables were chosen based on factors that are known to influence treatment retention, and health services use from the literature [40–43].

Age and sex are routinely collected variables both at Benbowpka and in administrative data in Ontario. Age was collected and categorized into four groups, 18–34, 35–54, 55–64 and over 65 years of age. This categorization has been used in several studies and was chosen to align with influential provincial reports on substance use [44].

We collected two variables related to Indigeneity for this study, self-identified Indigenous and Indigenous status. Indigenous status is the legal standing of a person who is registered under the Indian Act in Canada. In Canada, the term Indigenous comprises First Nations, Inuit and Métis peoples. It is an important distinction because a registered Indigenous person in Canada has access to certain benefits and is eligible for a range of federal and provincial or territorial programs and services.

We also collected information related to rurality and geographical location due to the well-known association between health and location of residence in Ontario [45–48]. At the time of the study, Local Health Integration Networks (LHIN) were regional planning entities that plan and administer funding for health services across 14 defined geographic areas of Ontario. We defined northern Ontario with LHINs 13 and 14 (North East and North West LHIN) and the remainder (i.e., LHINs 1-12) to define southern Ontario regions. We used the Statistics Canada Rural and Small Town definition to distinguish between rural and urban areas [49]. To create the income quintile variable, income quintiles were determined using neighborhood-level metrics (postal codes) from census data. Income is classified into five categories in the “master list”: 1 (lowest), 2, 3, 4, 5 (highest).

Program completion was our primary outcome. Secondary outcomes were evaluated at two time points: 6 months before entering residential treatment and 6 months after completing residential treatment. The secondary outcomes included the number of hospitalizations and emergency department visits for any reason and for substance-related issues (with ICD diagnostic codes: F10-19, F55, F63.0), number of mental health physician visits and number of primary care physician visits. Billing codes for physician visits are listed in Additional file 1. All outcomes, including health care utilization, were assessed for all patients.

### Statistical analysis

**Fig. 1:**
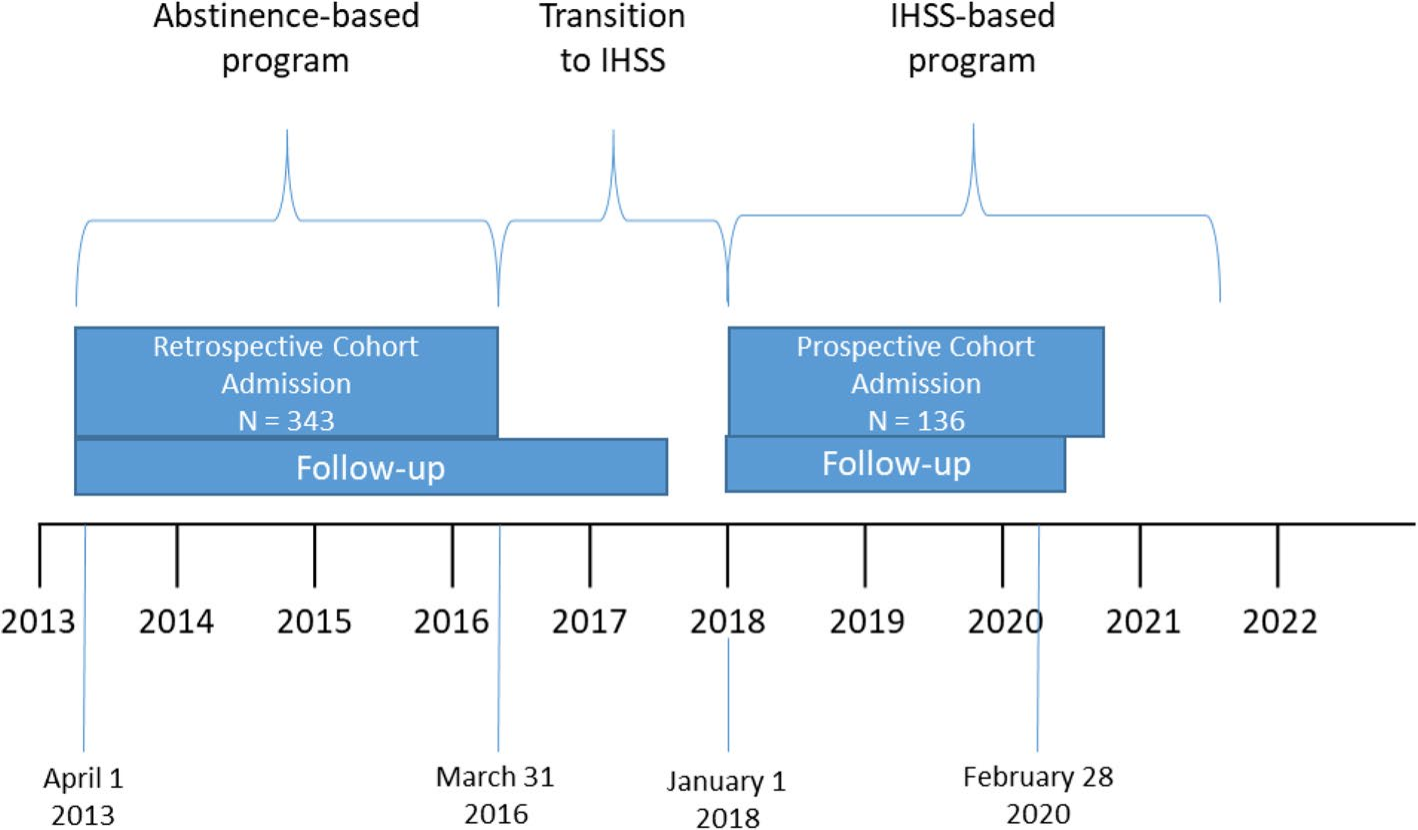
Project timeline

We analyzed anonymized data for patients who attended the Benbowopka Residential Treatment Centre in the no IHSS and IHSS study windows (see Fig. 1). A descriptive analysis was carried out to identify differences in patient characteristics between treatment groups. A Chi-square test was used, and the significance level was set at 0.05. A logistic regression model was used to evaluate the association between IHSS and program completion. We calculated odds ratios (OR) and adjusted ORs for baseline covariates and reported a 95% confidence interval for significance.

The final model was performed using PROC GEN-MOD (generalized estimating equation (GEE) regression model using exchangeable working covariate matrix and negative binomial regression) to evaluate health service use (including primary care visits, ED visits overall and for substance use, hospitalizations and mental health visits), with independent variables of IHSS versus no IHSS at two time points (time 1: 6 months before and time 2: 6 months after residential treatment), and baseline covariates included. The exponent of the point estimates from the GEE analysis was used to calculate odds ratios and 95% CI. All analyses were executed using SAS Enterprise Guide 7.1.

### Data governance and ethical considerations

Anonymized data collected at Benbowopka was linked in accordance with data-sharing agreements between ICES and the North Shore Tribal Council (Mamaweswen). We respected the Tri-Council Policy Statement, Chapter 9, highlighting the importance of engaging with First Nations throughout all phases of the research process. In addition, we honoured Indigenous knowledge by engaging with Elders and the Mamaweswen Council. All parties involved in the study respected the First Nations principles of OCAP® (First Nations Centre, 2007) by ensuring that the Mamaweswen Council maintained ownership of the data and control of the analysis and dissemination process. This research was in keeping with the Canadian Institutes of Health Research (2011) Guidelines for Research Involving Aboriginal People and the Tri-Council Policy Statement for Ethical Conduct for Research Involving Humans (Canadian Institutes of Health Research et al. 2011). The study received approval from Laurentian University’s Ethics Board in May 2017.

## Results

### Patient characteristics

A total of 266 residential treatment patients in the no-IHSS group and 136 in the IHSS group were included in the study. We observed patients’ health service use using administrative data 6 months before starting the residential treatment program and 6 months after completing the program regardless of completion of the 28-day program. There were no significant differences in age, sex, self-reported Indigeneity, on reserve, rural or northern status of patients between the no-IHSS and IHSS groups. However, there was a higher proportion of status First Nation patients in the no IHSS group (83.8% versus 73.5%, with 42 patients (10%) with missing data regarding First Nation status). A small but statistically significant difference in the neighbourhood income quintile (obtained from ICES data linkage) was seen with a higher proportion of patients in the lower-income quintile in the no-IHSS group and a high proportion of patients in the higher income quintile in the IHSS group was observed. At the time of enrollment, a higher proportion of patients in the IHSS group reported problematic use of opioids and stimulants (opioids: 16.2% versus 6.2; stimulants: 31.2% versus 6.2%) (Table 1).

**Table 1:** IHSS and no-IHSS group characteristics

Characteristics	No IHSS *n* = 266		IHSS *n = 136*		*P*-value
	*n*	*%*	*n*	*%*	
**Program Complete**	*183*	*68.8*	*102*	*75.0*	0.2
**Age at intake**					0.63
*18-34*	162	60.9	81	59.6	
*35-54*	89	33.5	44	32.4	
*55-64*	14	5.3	11	8.1	
*65+*	< 10	< 10	< 10	< 10	
**Sex (F)**					0.71
*Female*	118	44.4	63	46.3	
*Male*	148	55.6	73	53.7	
**Indigenous**					0.29
*Yes*	224	84.2	100	73.5	
*No*	38	14.3	23	16.9	
*Unknown*	< 10	< 10	< 10	< 10	
**Status First Nation (Missing*n*** **= 42)**					< 0.05
*Yes*	223	99.5	100	73.5	
*No*	< 10	< 10	34	25.0	
*Unknown*	< 10	< 10	< 10	< 10	
**On reserve (Missing = 141)**					0.03
*On reserve*	106	84.8	51	37.5	
*Off reserve*	11	8.8	84	61.8	
*Unknown*	8	2.4	< 10	< 10	
**Rural**	129	48.5	54	39.7	0.11
**North**	221	83.1	106	77.9	0.2
**Primary Substance at intake (Missing = 8)**					< 0.05
*Alcohol*	222	86.0	69	50.7	
*Cannabis*	6	2.3	< 10	< 10	
*Opioids*	14	5.4	22	16.2	
*Stimulants*	16	6.2	41	31.2	
**Income Quintile (Missing = 9)**					< 0.05
*1 (lowest)*	135	*52.5*	63	46.3	
*2*	51	*19.8*	22	16.2	
*3*	43	*16.7*	18	13.2	
*4*	13	*5.1*	22	16.2	
*5*	*15*	*5.8*	9	6.6	
Prior OAT	9	*3.4*	22	16.2	< 0.05

^a^Unknown - patient reported their status as unknown

^b^Missing - no data was collected

### Outcome results

A total of 183 (68.8%) patients from the no-IHSS group completed the program versus 102 (75.0%) from the IHSS patients. After adjusting for patient characteristics including age, sex, Indigenous, Status First Nation, on reserve, rural, north, primary substance, and income. IHSS was associated with a 95% improvement in program completion (Adjusted Odds Ratio (OR,1.95 95% Confidence Interval (95% CI), 1.02-3.70).

**Fig. 2:**
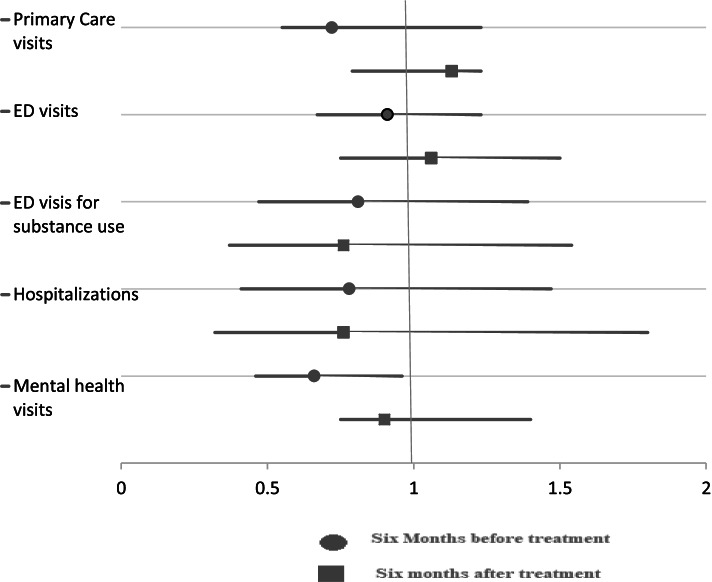
Impact of IHSS versus no-IHSS on health system outcomes 6 months before and 6 months after residential treatment (Odds ratio and 95% confidence intervals)

Table 2 and Fig. 2 results show that the IHSS patients had lower health service usage at time 1 (6 months before residential treatment) and 2 (6 months after residential treatment), with the exception of mental health physician visits (time 1: mean of 2.30 (standard deviation (STD) 5.12) and time 2: 1.62 (STD 4.07) in the IHSS group versus time 1: 0.91 (STD 1.48) and time 2: 0.74 (STD 2.10) in the no-IHSS group). There was no significant association between IHSS patients’ health service use at time 1 or time 2. Primary care visits time 1: aOR 0.55, 95%CI 0.72-1.13, time 2: aOR 1.13, 95%CI 0.79-1.23; ED visits overall time 1: aOR 0.91, 95%CI 0.67-1.23, time 2: aOR 1.06, 95%CI 0.75-1.50; ED visits for substance use time 1: aOR 0.81, 95%CI 0.47-1.39, time 2: aOR 0.79, 95%CI 0.37-1.54; Hospitalizations time 1: aOR 0.78, 95%CI 0.41-1.47, time 2: aOR 0.76, 95%CI 0.32-1.80; Mental health visits time 1: aOR 0.66, 95%CI 0.46-0.96, time 2: aOR 0.92 95%CI 0.7-1.40.

**Table 2:** Impact of IHSS versus no-IHSS on health system outcomes 6 months before and 6 months after residential treatment

	6 months before			6 months after		
	Mean (Std)	OR (95%CI)	aOR	Mean (Std)	OR (95%CI)	aOR
**Primary Care visits**						
IHSS	1.65 (2.43)	1.01 (0.90–1.13)	0.55 (0.72–1.23)	1.08 (1.94)	1.10 (0.78–1.52)	1.13 (0.79–1.23)
No IHSS (reference)	1.73 (2.24)			1.17 (1.99)		
**ED visits for any reason**						
IHSS	1.82 (2.60)	0.98 (0.75–1.28)	0.91 (0.67–1.23)	1.40 (2.50)	1.07 (0.77–1.50)	1.06 (0.75–1.50)
No IHSS (reference)	1.88 (2.6)			1.50 (2.80)		
**ED visits for substance use**						
IHSS	0.35 (1.01)	0.94 (0.59–1.48)	0.81 (0.47–1.39)	0.30 (1.00)	0.89 (0.49–1.73)	0.76 (0.37–1.54)
No IHSS (reference)	0.47 (1.20)			0.30 (1.20)		
**Hospitalizations**						
IHSS	0.16 (0.49)	1.25 (0.73–2.15)	0.78 (0.41–1.47)	0.17 (0.65)	0.77 (0.28–1.83)	0.76 (0.32–1.80)
No IHSS (reference)	0.12 (0.38)			0.12 (0.62)		
**Mental health visits**						
IHSS	2.30 (5.12)	0.88 (0.15–4.92)	0.66 (0.46–0.96)	1.62 (4.07)	1.64 (0.16–6.56)	0.92 (0.75–1.40)
No IHSS (reference)	0.91 (1.48)			0.74 (2.10)		

*ED* Emergency department, *STD* Standard deviation, *OR* Odds ratio, *aOR* Adjusted odds ratio, *95%CI* 95% confidence interval

## Discussion

Drawing on linked program data and longitudinal administrative data of patients from the Benbowopka residential treatment program, after adjusting for completion rates for patient characteristics, the IHSS group has better retention. However, there were no statistically significant differences in health service use, except for higher mental health physician visits.

Before the implementation of this research, the Benbowopka residential treatment program functioned as an abstinence-based program with the inclusion of traditional healing practices. We observed changes in patient characteristics after IHSS implementation (more off-reserve, more OUD, more stimulants). Such characteristics have been shown to be associated with a higher likelihood of dropping out of treatment. Therefore, the IHSS group had better retention when we adjusted for baseline covariates. The improved program completion rates are aligned with the original hypothesis and consistent with other studies that have demonstrated that providing culturally competent services combined with evidence-based interventions can improve health outcomes, increase clinical and support staff efficiency, and result in greater client satisfaction with services [50]. The Community Holistic Circle Healing (CHCH) process in Hollow Water, established in 1985, provides another example of successful community-based treatment for Indigenous people. Many elders and healers have affirmed that this community has the most mature healing process in Canada and addresses the needs of both sexual assault victims and victimizers and SUD (Buller 2013). Buller, 2013 writes, “Valdie Seymour and Berma Bushie are two of the founders from a First Nation of 700 people on the Eastern shore of Lake Winnipeg. In the mid-1980s, Hollow Water discovered a solution to sexual assault and SUD in the power of healing circles. They began with a resource team of 24, wherein the members of which openly shared their painful personal stories with each other” (p. 293). A few case studies and research reports incorporate healing rituals into conventional counselling and treatment interventions [2, 5, 13, 20, 51]. Scholars, traditional healers, and Elders encourage and utilize integration; however, some feel that these approaches require additional scientific evidence to support their use [13, 14, 20, 37, 52, 53].

In this study, when evaluating health service use, we observed no significant differences in outcomes 6 months before and 6 months after residential treatment between no-IHSS and IHSS groups. It is important to note that recruitment was from January 1, 2018, to March 31, 2020. The acute care and ER utilization of the subjects recruited from October 1, 2019, to March 31, 2020, may have been impacted by the COVID pandemic. However, the direction of this impact is unclear. For the general population, ER and acute care use was reduced during the early months of the pandemic. However, during this same period, the use of fentanyl and methamphetamine increased substantially among individuals with substance use disorders in Ontario (cite our two papers on this). Thus, the last portion of patients in the IHSS group might have had either increased or decreased use of ER and Acute care during the first 6 months of the COVID pandemic.

It is important to mention that the rates of health service use were consistently lower in this study population compared to rates shown in other studies in similar cohorts [42]. For instance, the mean ED visits in this study were approximately 1.5 visits in 6 months compared to a recent cohort study on people with opioid use disorder in Ontario, in which the mean ED visits were approximately 25 visits in 1 year [42]. Differences between this study and others in the rates of health service use may reflect the high proportion of Indigenous people in our cohort and the common discrimination in the health care system that directly negatively impacts health and well-being. In other settings, we see reduced primary healthcare utilization and concurrent increased tertiary healthcare utilization (particularly, potentially preventable hospitalizations) among Indigenous people compared to non-Indigenous people. Indigenous people report very unpleasant experiences of discrimination, which is considered a root cause of the health inequalities among Indigenous people [5, 15, 16, 27]. Experiences of discrimination and racism are prevalent in many treatment settings due to a lack of trauma-informed care and lack of knowledge. The types of discrimination often reported include abusive treatment, stereotyping, and a lack of quality in the care provided, which discourage Indigenous people from accessing care. Other ways that discrimination is displayed are receiving people in an unwelcoming environment, reinforcing stereotyping and stigma, and offering a practice informed by racism [5, 15, 16, 27].

To our knowledge, this is the only recent study that has evaluated health service use before and after Indigenous residential treatment and the only study assessing the impact of indigenous healing practices on health service use rates. However, the effect on ED visit numbers has been studied in other settings. For instance, in a cohort study evaluating health service outcomes comparing patients receiving mental health services and OAT to OAT alone, those receiving concurrent mental health and OAT services had higher rates of substance-related ED visits [54]. The higher rate may be related to patients’ higher health service needs with multiple comorbidities or limited access to sufficient, appropriate or specialized services in the community to prevent the need for acute care services [42, 55–57]. Future research should be undertaken to further explore how Indigenous residential treatment effectively improves health service outcomes, particularly in the context of trauma-informed practices in Indigenous communities.

Some limitations in the current study require consideration. First, recall and social-desirability bias could be present when using self-reported data, especially for socially stigmatized and criminalized behaviors (such as primary substance use in this study). Second, this study utilized a very specific sample of patients in a residential treatment program in northern Ontario, limiting the generalizability of the findings. Third, the follow-up time to evaluate health service use data was shorter than anticipated because of the program’s suspension due to COVID-19 leading to interruptions in data collection. The lack of significant associations may have been different if recruitment times were longer leading to a larger IHSS sample size. Fourth, although we considered several factors associated with health service use, there is potential for unmeasured confounding. Lastly, it is important to note that when using secondary administrative health data there is potential for data entry errors, incomplete or inaccurate capture of data, and missing values.

## Conclusion

In summary, the IHSS group has some changes in client characteristics (more off-reserve, more prior OAT, more patients with problematic opioid and stimulant use) associated with a higher likelihood of dropping out of treatment. But despite this, the treatment completion rates overall are similar. So when we adjust the completion rates for patient characteristics, the IHSS group has better retention. The result from this study clearly indicates that clients benefitted from both the evidence-based treatment model, Seeking Safety, and traditional healing practices with regards to completing the program. The atrocities, oppression, and multiple losses the Indigenous people endured in Canada, motivated Elders, leaders, and communities to embrace their strength, resilience and to move forward toward healing practices and ceremonies to reclaim their dignity and traditional healing practices [3, 14, 53]. Our results suggest a need to develop more comprehensive treatment strategies specific to Indigenous people who experience trauma and substance use to maximize the benefits of treatment.

## Supplementary Information

**Additional file 1.** Ontario Health Insurance Plan Fee Codes.

## Data Availability

The dataset from this study is held securely in coded form at ICES. While legal data sharing agreements between ICES and data providers (e.g., healthcare organizations and government) prohibit ICES from making the dataset publicly available, access may be granted to those who meet pre-specified criteria for confidential access, available at www.ices.on.ca/DAS (email: das@ices.on.ca). The full dataset creation plan and underlying analytic code are available from the authors upon request, understanding that the computer programs may rely upon coding templates or macros that are unique to ICES and are therefore either inaccessible or may require modification.
