# Supplementary material for: Community trial evaluating the integration of Indigenous healing practices and a harm reduction approach with principles of seeking safety in an Indigenous residential treatment program in northern Ontario
Source: BMC Health Serv Res. 2022 Aug 16;22:1045. doi: 10.1186/s12913-022-08406-3 (PMC9381149; doi:10.1186/s12913-022-08406-3)
Supplement: Supplementary file 1 — Additional file 1. Ontario Health Insurance Plan Fee Codes. [file 12913_2022_8406_MOESM1_ESM.docx]

Additional file 1: Ontario Health Insurance Plan Fee Codes

| ***Fee Code*** | ***Description*** |
| --- | --- |
| A001 | MINOR ASSESS.-F.P./G.P. |
| A002 | Family Practice & Practice in General - Enhanced 18 month well baby visit |
| A003 | GEN. ASSESS. -F.P./G.P. |
| A007 | INTERMED.ASSESS/WELL BABY CARE-F.P./G.P./PAED. |
| A903 | GEN/FAM PRACT-PRE-DENTAL/OPER.ASSESS LIMIT 2 PER YEAR/PT |
| E075 | GERIATRIC GENERAL ASSESSMENT PREMIUM |
| G212 | D./T. PROC.-ALLERGY-HYPOSENSITIZATION INJECTION PLUS BASIC |
| G271 | D./T. PROC.-CARDIOV.-ANTICOAGULANT SUPERVISION |
| G372 | D./T. PROC.-INJECTIONS-INTRADERMAL/MUSCULAR ETC. EA. ADD. |
| G373 | D./T. PROC.-INJ. INTRADERMAL/MUSC. BASIC FEE (SHICK TEST) |
| G365 | D./T. PROC.-GYNAECOLOGY-PAPANICOLAOU SMEAR |
| G538 | D&T IMMUNIZATION-WITH VISIT, EACH INJECT. |
| G539 | Injection of unspecified agent - sole reason (first injection) |
| G590 | INFLUENZA AGENT +VISIT |
| G591 | Injection of influenza agent - sole reason |
| K005 | INDIVIDUAL CARE PER 1/2 HR |
| K013 | COUNSELLING-ONE OR MORE PEOPLE-PER 1/2HR. |
| K017 | ANNUAL HEALTH EXAM-CHILD AFT. 2ND BIRTHDAY. |
| P004 | OBS.-PRENATAL CARE-MINOR PRENATAL ASSESS.-SUBSEQ.PRENAT.VIS. |
| K130 | Periodic health visit - adolescent |
| K131 | Periodic health visit - adult aged 18 to 64 inclusive |
| K132 | Periodic health visit - adult 65 years of age and older |
| A261 | MINOR ASSESS.-PAED. |
| A268 | Paediatrics - Enhanced 18 month well baby visit |
| K267 | ANNUAL HEALTH EXAM-CHILD-AFT. 2ND BIRTHDAY PAED. |
| K269 | ANNUAL HEALTH EXAM-PAEDIATRICS-ADOLESCENT-OFFICE |
